# Mechanism and effect of γ-butyrolactone solvent vapor post-annealing on the performance of a mesoporous perovskite solar cell

**DOI:** 10.1039/c7ra10695e

**Published:** 2018-01-03

**Authors:** Jun Luo, Ren Zheng Qiu, Zhi Sheng Yang, Yan Xiang Wang, Qi Feng Zhang

**Affiliations:** School of Materials Science and Engineering, Jingdezhen Ceramic Institute Jingdezhen 333403 China yxwang72@163.com; Department of Electrical and Computer Engineering, North Dakota State University Fargo ND 58108 USA qifeng.zhang@ndsu.edu

## Abstract

In this paper, γ-butyrolactone (GBL) solvent vapor post-annealing (SVPA) on CH_3_NH_3_PbI_3_ thin films is reported, aiming to improve the complete transformation of PbI_2_ and increase the grain size of the CH_3_NH_3_PbI_3_ crystal, thus boosting the performance of mesoporous CH_3_NH_3_PbI_3_ perovskite solar cells (PSCs). The influence of GBL SVPA on the microstructure of perovskite layers and performance of PSCs was studied. The short circuit current density (*J*_sc_) of the devices significantly increased, yielding a high efficiency of 16.58%, which was 27.05% higher than that of thermally annealed films. A model was derived to explain the effect of GBL SVPA on PSCs. The perovskite films prepared by this method present several advantages such as complete transformation of PbI_2_ to CH_3_NH_3_PbI_3_, high crystallinity, large grain size, and fewer grain boundaries than those prepared without GBL SVPA. This improvement is beneficial for charge dissociation and transport in hybrid photovoltaic devices.

## Abstract 2

γ-Butyrolactone was used to post-anneal perovskite films and better performance of cells was achieved; a mechanism was derived.

## Graphical abstract

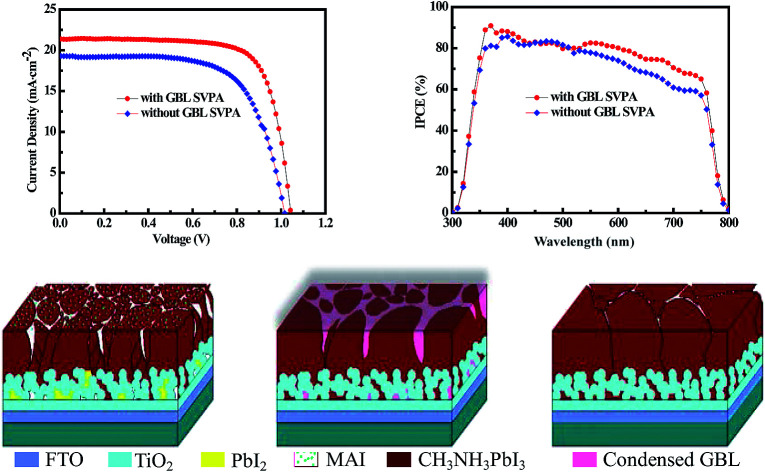

## Introduction

In recent years, organometal halide perovskite solar cells (PSCs), which are considered to be the most promising next-generation solar cells, have been extensively investigated.^1,2^ In addition to their intense broadband absorption,^3^ this type of PSC also possesses small exciton binding energies (around 50 meV at room temperature),^4,5^ long charge carrier diffusion lengths (100–1000 nm) and lifetimes (∼100 ns),^6^ good ambipolar charge mobilities, and low cost.^7^ PSCs have attracted worldwide attention due to these merits. Moreover, organometal halide perovskite materials can be solution-processed at low temperatures. With the development of organometal halide PSCs, the power conversion efficiency (PCE) of PSCs has increased from 3.8%^8^ to >21%.^9^

As a polycrystalline thin film, the optoelectronic properties of perovskite films and device performance highly depend on morphology such as crystallinity and grain size.^10^ The groups of Huang and Mohite have respectively demonstrated that the diffusion length and carrier mobility can be significantly improved in large grained perovskite films (over 3 μm).^11–13^ This suggests that ideal perovskite films for solar cells should consist of grains as large as possible. However, solution-processed perovskite films usually have relatively small grain sizes (within a couple of hundred nanometers (nm)) due to the quick reaction of lead iodide (PbI_2_) and methyl-ammonium iodide (MAI), and the quick crystallization of these perovskite materials (MAPbI_3_). The small grain size of MAPbI_3_ has more grain boundaries, which increases charge recombination and results in a decrease in PCE. A great deal of efforts have been made to control the morphology (larger grain size and better crystallinity) of the perovskite films by varying precursor concentrations^14,15^ or ratios,^16,17^ adjusting the annealing conditions,^18,19^ and using additives.^20–22^ The solvent or vapor assisted process is an effective method to optimize the quality of perovskite films.^14,23–29^ Solvents such as *N*,*N*-dimethylformamide (DMF) have been successfully applied in solvent-assisted processes.^23,30^ Introducing DMF vapor during the annealing process provides the wet environment for the precursor ions and molecules to diffuse a long distance, resulting in growth of large sized grains. In addition to the dissolving solvent, dropping a non-dissolving solvent (such as toluene, diethyl ether, *etc.*) into a perovskite precursor film during the spin coating process has also been used to produce highly crystalline uniform perovskite films.^14,17,31^

The selected non-dissolving solvent, which does not dissolve the perovskite materials and is miscible with other solvents (added to dissolve PbI_2_ and MAI), is dripped on the substrate where the perovskite is deposited during spin-coating. Subsequently, a stable intermediate phase is formed *via* an intercalation process during the drop-wise application of a non-dissolving solvent. It is a decisive factor in retarding the rapid reaction between MAI and PbI_2_, which enables the formation of a highly uniform and dense film. Eventually, perovskite can be obtained after thermal annealing. However, these methods are difficult to control accurately.

The perovskite film will be eroded if excessive solvent is added. Similarly, the homogeneous perovskite film will not be obtained if the non-dissolving solvent is not dropped at the accurate time.^31^ In addition, it has been difficult to extend these operations to large area production. Therefore, exploring a simple and effective method to produce high quality perovskite films is required.

The solvent vapor post-annealing (SVPA) process is different from the solvent- or vapor-assisted process. The SVPA process involves heating the prepared perovskite films in some solvent vapor for a specific time.^32,33^ The SVPA process has been widely used to fabricate organic thin films and solar cells to control the morphology.^34,35^ During the SVPA process, solvent molecules are absorbed in the thin films. The absorbed solvent may decrease the diffusive energy barrier and promote the rearrangements of grains,^34^ which will improve the crystallinity and carrier mobility of the perovskite films. In 2014, Huang first reported that the SVPA process is an effective method to increase the grain size and carrier diffusion lengths of trihalide perovskite materials.^33^ They found that the average grain size of the CH_3_NH_3_PbI_3_ films after the SVPA process increased to 1 μm, which was comparable to the film thickness, while the maximum grain size in thermally annealed films was only around 260 nm. In 2015, Liu systematically studied the influence of different SVPA atmospheres on perovskite films including N_2_, H_2_O, DMF, γ-butyrolactone (GBL), and dimethyl sulfoxide (DMSO). They found that DMSO was the best solvent.^32^ Fang *et al.* reported high quality CH_3_NH_3_PbI_3−*x*_Cl_*x*_ perovskite films using chlorobenzene (CB) vapor post-annealing.^36^ They found that this method had a positive effect on the interfacial contact between the perovskite film and the upper PCBM film. Hybrid PSCs with planar heterojunctions fabricated by this method demonstrated a reproducible optimal PCE of 14.79% and an average PCE of 13.40%, which were better than those when thermally annealed.

Compared with the prevailing anti-solvent dripping method—which needs precise control of the dripping timing—SVPA is more compatible and reproducible for preparing large-area and high-quality perovskite thin films, opening up opportunities for the development of high performance perovskite solar cells and other optoelectronic devices.

To the best of our knowledge, there are very few articles reporting the SVPA of perovskite films. Only a few solvents were used such as phenyltrichlorosilane (PTS), octadecyltrichlorosilane (OTS),^37^ water,^38^ alcohol,^39^ dimethylsulfoxide (DMSO),^40^ and DMF.^41–45^ The most commonly used solvents are DMSO and DMF. It is easy to form coordination complexes, accompanied by volume expansion when using DMSO or DMF as solvent for vapor post-annealing to prepare perovskite films.^17,46^ Moreover, residual DMSO or DMF may form pinholes and destroy the perovskite layer causing volume shrinkage. Moreover, only a few articles demonstrated the morphology and performance analysis of the corresponding photovoltaic devices with and without SVPA. There are no other related reports on the mechanism and effect of SVPA on the morphological characteristics and revolutions of the perovskite films. A detailed study is helpful to break the bottleneck and obtain better performances in large grain PSCs.

GBL is a low solubility solvent for CH_3_NH_3_PbI_3_. It is difficult to form coordination complexes in GBL solution while single crystals of perovskite can be formed in GBL solution.^47^ When the temperature of GBL solution is near 60 °C, the solubility of CH_3_NH_3_PbI_3_ is highest. This indicates that the GBL solution is a good candidate for SVPA to obtain high quality perovskite films at low temperature, which will reduce the cost and be suitable for flexible thin-film solar cells.

Inspired by existing related studies, we adopted GBL for post-annealing treatment of perovskite films. In this study, we investigated the effect of GBL solvent vapor post-annealing on the characteristics of perovskite films and performances of the corresponding PSCs. Moreover, we proposed the mechanism of GBL SVPA. We found that larger grain size, better crystallinity and complete reaction of PbI_2_ with MAI are the main factors that led to improved photoelectric performance. Eventually, PCE of 16.58% was achieved.

## Experimental section

### Materials

Methylammonium iodide (CH_3_NH_3_I, 99.8%, Dyesol), lead iodide (PbI_2_, 99.999%, Sigma), *N*,*N*-dimethylformamide (DMF, 99.9%, Aladdin), dimethyl sulfoxide (DMSO, 99.9%, Aladdin), chlorobenzene (99.9%, Aladdin), 1-butanol (99.6%, Aladdin), lithium bis(trifluoromethanesulfonyl)imide (Li-TFSI, 99%, Aladdin), γ-butyrolactone (GBL, 99%, Aladdin), isopropanol (99.7%, Sinopharm Chemical Reagent Co., Ltd), titanium diisopropoxide bis(acetylacetonate) (75 wt% in isopropanol, Sigma Aldrich), TiCl_4_ (>98%, Sigma Aldrich), and spiro-MeOTAD (>99.5%, Lumtec) were used as hole receptor without further purification.

### Perovskite film and solar cell fabrication

F-doped SnO_2_ (FTO, NSG, TEC A7) substrates were cleaned with a sequence of detergent, deionized water, acetone, and isopropanol for 15 min in an ultrasonic bath. The precleaned FTO substrates were dried under a nitrogen stream and subjected to ultraviolet ozone treatment for 20 min.

The TiO_2_ blocking layer (BL) was spin-coated on the FTO substrate at 2000 rpm for 30 s using a solution of 0.15 M titanium diisopropoxide bis(acetylacetonate) in 1-butanol, which was heated at 135 °C for 10 min. After cooling to room temperature, the spin-coating process was repeated to obtain a TiO_2_ BL with proper thickness. A mesoporous TiO_2_ layer composed of 20 nm nanoparticles was then prepared by spin-coating at 5000 rpm for 30 s using a commercial TiO_2_ paste (Dyesol 18NRT, Dyesol) diluted in ethanol (2 : 7 mass ratio weight ratio). The as-deposited TiO_2_ films were dried at 135 °C for 10 min, gradually heated to 500 °C in air, and finally baked at this temperature for 30 min to remove organic components. The electrodes were soaked in 40 mM TiCl_4_ aqueous solution at 70 °C for 30 min and then rinsed with deionized water followed by annealing at 500 °C for another 30 min. Finally, the films were subjected to UV/ozone treatment for 15 min.

PbI_2_ solution was prepared by dissolving 462 mg PbI_2_ in a mixed solvent of 74 μL DMSO and 700 μL DMF while stirring at 60 °C. Prior to spin-coating of PbI_2_, the PbI_2_ solution and mesoporous TiO_2_ thin film were heated at 105 °C. The PbI_2_ solution (80 μL) was spin-coated on the mesoporous TiO_2_ film at 5000 rpm for 30 s and 300 μL chlorobenzene was spin-coated again. After spinning, the film was immersed into MAI solution (10 mg mL^−1^ in isopropanol) for 10 min. The corresponding thin film was spin-coated at 3000 rpm again to dry the film. Finally, the complex film was annealed at 105 °C for 10 min in ambient air (relative humidity 40% at 25 °C).

**Scheme 1:**
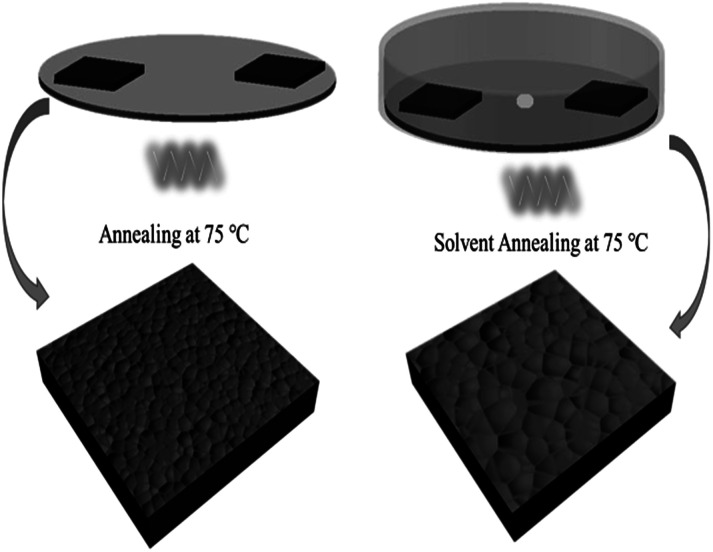
Schematic illustration of GBL-vapor solvent post-annealing process.

For the film treated with SVPA, the perovskite films were put on a hotplate, covered with a Petri dish, and annealed at 75 °C for about 30 min. GBL solvent (10 μL) was dropped at the center of the Petri dish so that GBL vapor could enter the Petri dish and form the GBL vapor atmosphere. The processing scheme for perovskite thin film formation using solvent vapor post-annealing methods is shown in Scheme 1. The stacking films were then annealed at 100 °C with or without GBL vapor for 1 h. The films without solvent annealing only went through thermal annealing and were used as control samples.

After annealing treatment with GBL solvent, a volume of 80 μL of 2,2′,7,7′-tetrakis(*N*,*N*-di-*p*-methoxyphenylamine)-9,9-spirobifluorene (spiro-MeOTAD) solution was spin-coated on the CH_3_NH_3_PbI_3_ perovskite layer at 3000 rpm for 30 s. The spiro-MeOTAD solution was prepared by dissolving 72.3 mg of spiro-MeOTAD in 1 mL of chlorobenzene, to which 28.8 μL of 4-*tert*-butyl pyridine and 17.5 μL of lithium bis(trifluoromethanesulfonyl)imide (Li-TFSI) solution (520 mg mL^−1^ in acetonitrile) were added. Finally, 80 nm thick gold was thermally evaporated on top of the device to form the back contact. The active area was fixed to 0.1 cm^2^ using a black mask.

### Characterization

The crystal structures of the samples were characterized using an X-ray diffraction (XRD) system (Bruker D8 Advance) with Cu-K_α_ (1.5406 Å). The morphologies of the samples were investigated by scanning electron microscopy (SEM, Hitachi S-4800). The energy conversion efficiencies of solar cells were evaluated under AM1.5 (100 mW cm^−2^) simulated sunlight (Newport, Serial 382, LampSBF178, Model 94023A). A power source meter (Keithley 2400) was used to measure the response of the solar cells. Incident photo-to-current conversion efficiencies (IPCE) of PSCs were measured by a solar cell quantum efficiency measurement system (Newport, 150 W xenon lamp, with a CS260-USB-Q-MC-A monochromator and 2936-R power meter). Ultraviolet-visible absorption spectra were recorded on a spectrophotometer (PerkinElmer, Lambda 850) in the 400–1100 nm wavelength range at room temperature. All measurements of the solar cells were performed under ambient atmosphere at room temperature.

## Results and discussion

### Characterization of perovskite layer

**Fig. 1:**
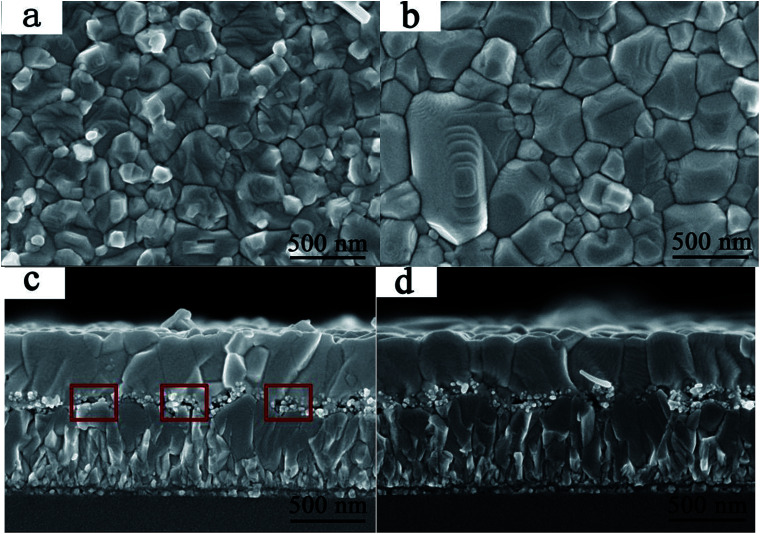
Top-view and cross-section SEM images of perovskite layers (a), (c) without GBL SVPA, and (b), (d) with GBL SVPA.

**Fig. 2:**
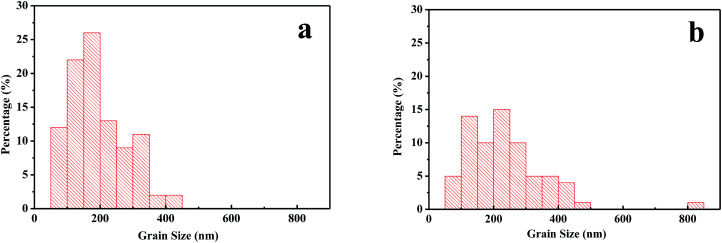
Grain size distributions of perovskite layers (a) without GBL SVPA (b) with GBL SVPA.

The influences of GBL SVPA on the morphology and crystal structure of the perovskite films were examined by SEM and XRD. Fig. 1 shows SEM images of perovskite films. The thermally annealed perovskite film exhibits smaller crystals and possesses many crystal boundaries (Fig. 1a). These boundaries will be trapping centers for exciton recombination and will reduce the *J*_sc_ and the PCE of the photovoltaic devices. In contrast, when GBL SVPA is introduced, the as-prepared perovskite films in Fig. 1b possess large crystallites and low densities of crystal boundaries, resulting in a surface morphology with higher homogeneity. Their in-plane grain size distributions charts were drawn using Image-Pro-Plus software (Fig. 2). The in-plane grain sizes of the perovskite layers without and with SVPA were 193 nm and 235 nm, respectively. In addition, cross-sectional SEM images of these devices (Fig. 1c and d) imply that the grains along the thickness of the device in the GBL SVPA perovskite film are also larger than those of the thermally annealed device. Fig. 1d shows that the grains of the GBL SVPA perovskite film penetrate the entire capping layer. Thus, there is no need to cross any grain boundary when the carriers are transported to the electrode, which greatly enhances the charge extraction process and reduces recombination to improve solar cell performance. In comparison, the photo-generated charges of the perovskite film without SVPA have to cross several grain boundaries during their transport in the out-of-plane direction before being collected by the electrodes. Moreover, Fig. 1c and d also indicate that GBL SVPA can improve the perovskite filling on mesoporous TiO_2_ layer and that most unfilled interspaces disappeared after GBL SVPA, which will enhance charge transport and the performance of mesoporous PSCs.^48^

**Fig. 3:**
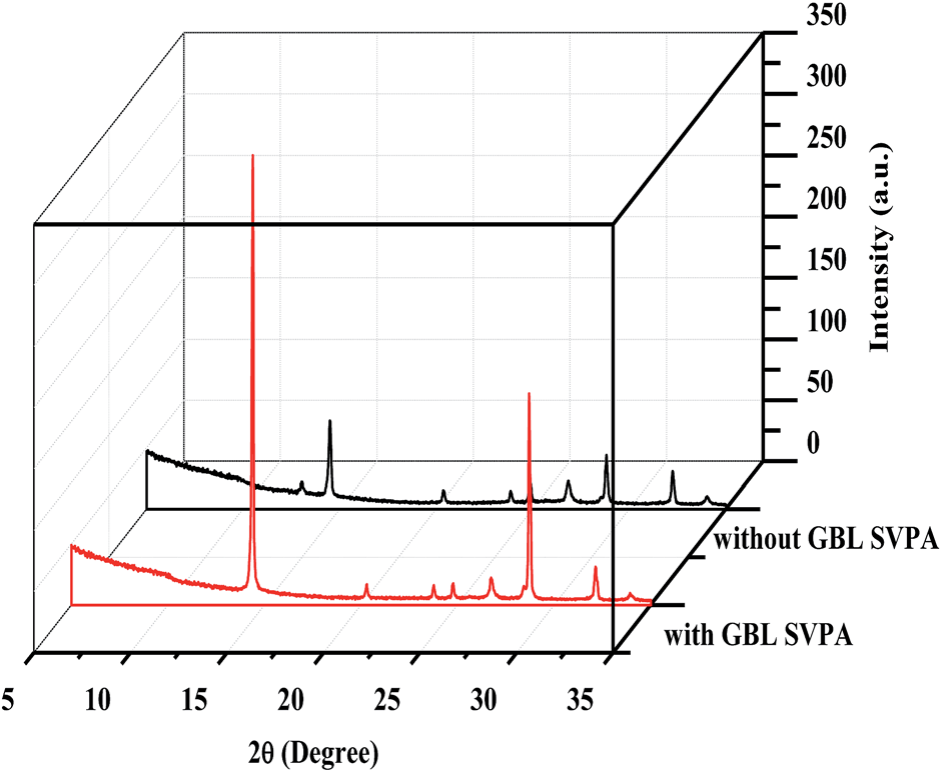
XRD patterns of perovskite layers.

**Scheme 2:**
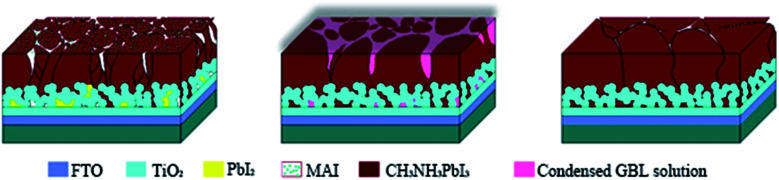
The mechanism of GBL SVPA on the perovskite films.

The XRD patterns are shown in Fig. 3. The XRD results indicate that they exhibit a tetragonal crystal structure. Peaks at 14.2°, 20.1°, 23.5°, 24.5°, 28.5° and 31.9° are diffracted by the (110), (020), (211), (202), (001), and (310) planes of CH_3_NH_3_PbI_3_, respectively. The peak at 26.6° is the diffraction of FTO. For the films after exposure to GBL vapor annealing, the two main peaks located at 14.2° and 28.5°—indexed to the (110) and (220) planes—became stronger. The stronger and sharper diffraction peaks of films annealed in solvent vapor certify the improved crystallinity of the perovskite films annealed in GBL vapor. In addition, for the film without GBL SVPA a peak at 12.7° related to PbI_2_ (001) is also observed, which indicates the presence of residual PbI_2_. The results also indicate that PbI_2_ is not completely transformed to CH_3_NH_3_PbI_3_ without GBL SVPA. It has been shown that the outer CH_3_NH_3_PbI_3_ layer insulates PbI_2_ from contact with the MAI solution and PbI_2_ remains in the films without SVPA.^21,49^ After GBL SVPA, no PbI_2_ was detected and the diffraction intensity obviously increased. That could explain how GBL SVPA enhanced the transformation of PbI_2_ and induced the strong recrystallization of CH_3_NH_3_PbI_3_. On the basis of the detailed investigation of the microstructures and XRD results of perovskite films, the effect and mechanism of GBL SVPA on the perovskite films were unveiled. A model (shown schematically in Scheme 2) is proposed. The low surface tension of GBL can wet perovskite well. According to the Kelvin equation (eqn (1)), the saturated vapor pressure decreases with the shrinking radius of the capillary and the liquid state solvent can easily condense in the capillary. The micro-cracks between grains and pinholes in the perovskite films act like capillaries; thus, liquid solvent can condense in microcracks or pinholes even when the vapor pressure of solvent is lower than the saturated vapor pressure. Once the grain boundary of the films is filled with GBL, perovskite can be easily dissolved in the polar GBL solvent. The solubility of the solid particle can be explained using eqn (2). According to this equation, the solubility increases with decrease in particle size; therefore, the small grains dissolve first. The atoms enter the liquid phase across the liquid–solid interface and then recrystallize in areas of larger grains with lower chemical potential, which leads to an increase in grain size and crystalline quality of the grains.

In this study, perovskite layers were spin-coated instead of washing with isopropanol after dipping in MAI solution for 10 min. Hence, there is residual MAI at the grain boundaries or on the surface. GBL solvent can also dissolve the residual MAI, then diffuse to the depth of perovskite films and react with the residual PbI_2._ The residual PbI_2_ would lead to decreased light absorption, photo-current generation, and increased charge accumulation.^50^ Volume expansion derived from the reaction of the residual PbI_2_ and MAI would fill the pores in the mesoporous layer, decreasing the porosity of the mesoporous layer, which is consistent with the SEM result.1
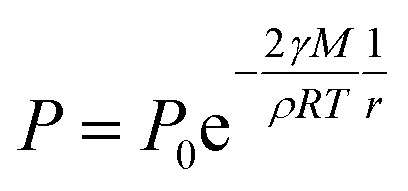
*P*: saturated vapor pressure of liquid in the wettable capillary, *P*_0_: saturated vapor pressure of planar liquid, *γ*: surface tension of liquid, *M*: relative molecular mass of liquid, *ρ*: density of liquid, *R*: gas constant, *T*: absolute temperature, and *r*: radius of capillary.2
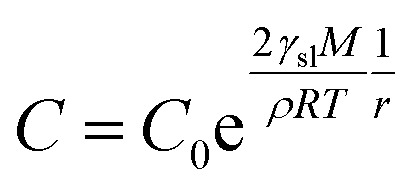
*C*: solubility of small particle, *C*_0_: solubility of bulk solid, *ρ*: density of solid, *γ*_sl_: tension of liquid–solid interface, *M*: relative molecular mass of solid, *R*: gas constant, *T*: absolute temperature, *r*: radius of small particle.

### Solar cell performance

**Fig. 4:**
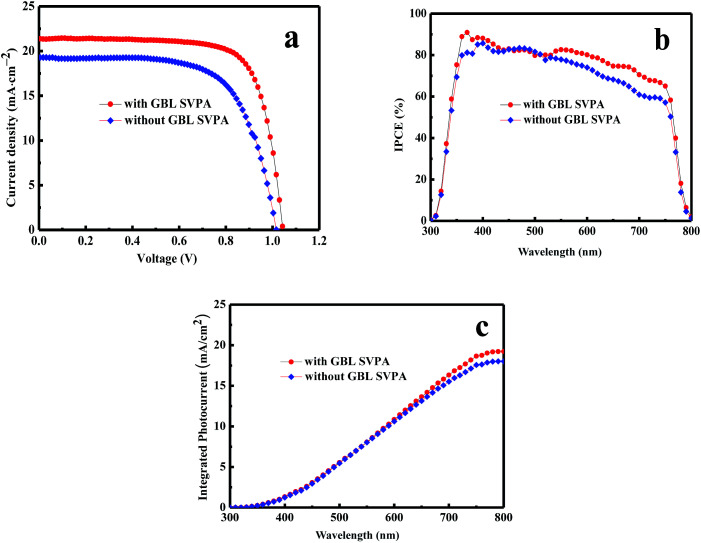
*J*–*V*, IPCE and integrated photocurrent curves of devices.

PSCs were fabricated to probe the effect of GBL SVPA on device performance. Fig. 4 contains the current density–voltage (*J*–*V*) and IPCE curves of the solar cell. The detailed photovoltaic parameters are summarized in Table 1. As expected, the GBL SVPA devices displayed better performance than those without GBL SVPA. The short circuit current density (*J*_sc_), open circuit voltage (*V*_oc_), fill factor (FF), and PCE of the device after GBL SVPA were higher than those of the device without GBL SVPA. *J*_sc_ increased from 19.3 mA cm^−2^ for the device without GBL SVPA to 21.3 mA cm^−2^ for the device with GBL SVPA. The PCE of the device with GBL SVPA reached 16.58%, which was 27.05% larger than the value of the device without GBL SVPA. The obviously improved *V*_oc_ from 1.01 V (device without GBL SVPA) to 1.04 V (device with GBL SVPA) means that the potential loss in the device is reduced. The *V*_oc_ in a photovoltaic device is determined by the quasi-Fermi level splitting of electrons and holes in the whole device under illumination, which is mainly affected by the occupation of available electronic states by photo-generated charge carriers in the perovskite layer. If there are many defects and recombination centers induced by structural and chemical disorder such as low crystallinity, grain boundaries, and random orientations, the occupation of the available electronic states in the perovskite layer will change, which can reduce the quasi-Fermi level splitting value. Thus, there will be a relatively small *V*_oc_. Since all devices were fabricated by the same procedure except for the perovskite layer annealing condition, the differences in *V*_oc_ between the devices should reflect the quality of the perovskite layer. A larger *V*_oc_ should result from a better perovskite quality.

**Table 1:** Photovoltaic parameters of PSCs

Sample	*V*_oc_ (V)	*J*_sc_ (mA cm^−2^)	FF (%)	PCE (%)
With GBL SVPA	1.04	21.30	0.75	16.58
Without GBL SVPA	1.01	19.30	0.67	13.05

The amounts of GBL solvent and SVPA time were varied in the preparation of perovskite film in our experiment. The results are summarized in Table 2. All devices were fabricated by the same procedure except for the variable amounts of GBL solvent and SVPA time in the preparation of perovskite film. We found that the device achieved best efficiency when the SVPA time was 30 min and the GBL solvent was 10 μL.

**Table 2:** The performance of PSCs fabricated at different condition

GBL solvent/SVPA time	*V*_oc_ (V)	*J*_sc_ (mA cm^−2^)	FF (%)	PCE (%)
0 μL/30 min	1.01	19.30	0.67	13.05
5 μL/30 min	1.02	20.15	0.73	15.06
10 μL/30 min	1.04	21.30	0.75	16.58
20 μL/30 min	0.99	21.11	0.71	14.99
10 μL/10 min	1.01	20.77	0.71	14.92
10 μL/20 min	1.03	21.00	0.73	15.78
10 μL/30 min	1.04	21.30	0.75	16.58
10 μL/60 min	1.00	20.88	0.71	14.91

**Fig. 5:**
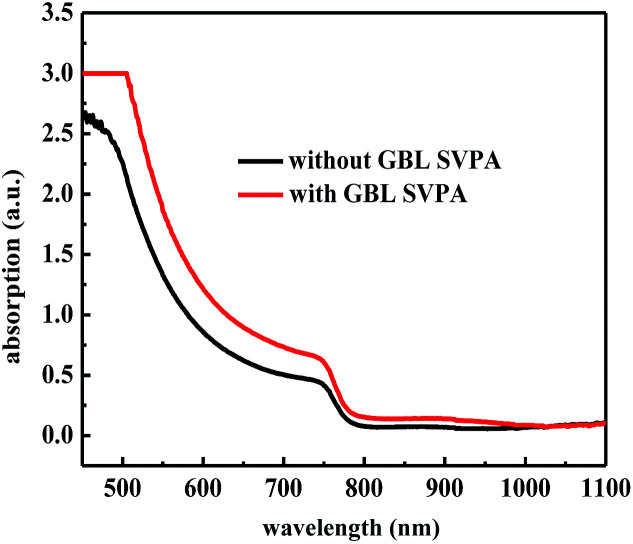
UV-vis absorption spectra of perovskite layers.

The performance improvement of the device can be attributed to the following two reasons. First is that the device with GBL SVPA has larger grain sizes and better crystal quality, which are expected to reduce the overall bulk defect density and hence suppress charge trapping and exciton recombination. The second is due to the complete reaction of PbI_2_ with MAI, which increases the amount of light absorbing perovskite material. The UV-vis absorption spectra of the perovskite layers were also measured (Fig. 5). Fig. 4b also shows representative IPCE curves for the devices. The curves begin to increase rapidly around 350 nm, which is related to the high optical absorption of the perovskite absorber. A higher IPCE was observed for the GBL SVPA device. The slightly higher IPCE of the GBL SVPA device from 400 nm to 700 nm is in agreement with the increase of *J*_sc_ associated with these devices. *J*_sc_ is calculated by integrating the IPCE spectrum based on eqn (3).^50^ The calculated current densities are 19.23 mA cm^−2^ and 18.01 mA cm^−2^ for the devices with and without GBL SVPA, respectively, which are close to the measured *J*_sc_ values.3
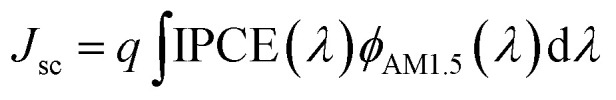
*J*_sc_: short circuit current, *q*: quantity of electric charge, IPCE (*λ*): the obtained IPCE profile as a function of wavelength (*λ*), and AM1.5 (*λ*): the solar spectral irradiance at a specific wavelength (*λ*).

**Fig. 6:**
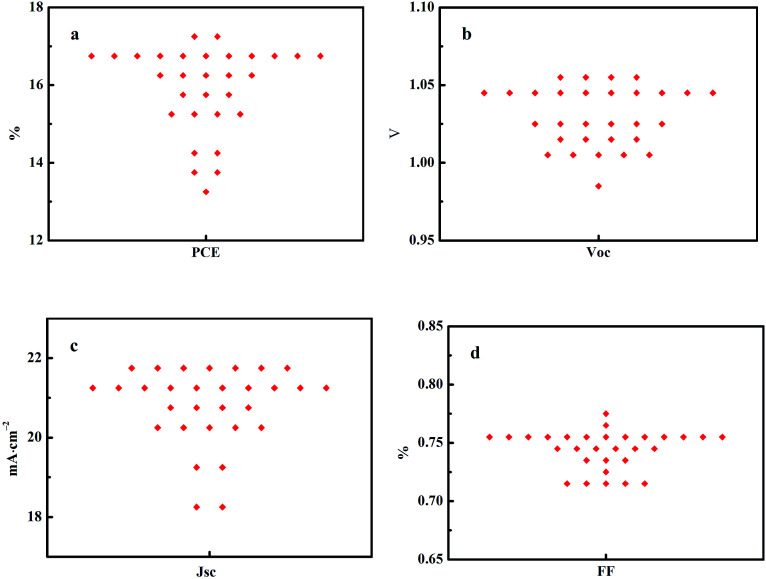
Statistics distribution chart of the performance of PSCs with GBL SVPA.

**Fig. 7:**
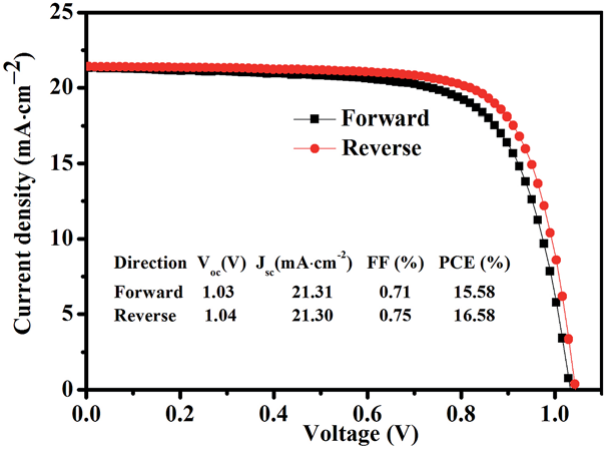
The performance of PSCs with GBL SVPA in both forward and reversed sweeping.

Fig. 6 shows statistics distribution charts of the performance of PSCs. As reflected in Fig. 6, the average *V*_oc_, *J*_sc_, FF and PCE were 1.03 ± 0.2 V, 20.76 ± 0.92 mA cm^−2^, 0.74 ± 0.02, and 15.93 ± 1.06% respectively. 30 cells were measured in total. Fig. 7 presents the performance of PSCs in both forward and reversed sweeping. As can be seen from Fig. 7, the reverse sweeping of the device has a slight advantage in the efficiency and fill factor.

## Conclusions

GBL SVPA was introduced to fabricate high quality CH_3_NH_3_PbI_3_ perovskite films. We have studied the effect of GBL SVPA on the microstructure and crystal quality of the grains of the perovskite layer and the photovoltaic performance of devices. A theoretical mechanism of GBL SVPA was also proposed based on the chemical physics of surfaces. After GBL SVPA at 75 °C, the corresponding perovskite layers had larger grain sizes, better crystal quality of the grains, and no detected residual PbI_2_. The energy loss in PCSs is derived from the non-radiative recombination of charges due to trap states on film surfaces, at grain boundaries, and at point defects (such as vacancies or interstitial defects) in the perovskite crystal lattice. Perovskite films with large crystallites and grain size would effectively remove these points, suppress energetic disorders, and enable improved performance of perovskite photovoltaic devices. The highest PCE of 16.58% was achieved when illuminated and tested under standard AM1.5 conditions.

## Conflicts of interest

There are no conflicts to declare.

## Supplementary Material

## References

[cit1] Wojciechowski K., Saliba M., Leijtens T., Abate A., Snaith H. J. (2013). Energy Environ. Sci..

[cit2] Burschka J., Pellet N., Moon S. J., Humphry-Baker R., Gao P., Nazeeruddin M. K., Grätzel M. (2013). Nature.

[cit3] Wolf S. D., Holovsky J., Moon S. J., Löper P., Niesen B., Ledinsky M., Haug F. J., Yum J. H., Ballif C. (2014). J. Phys. Chem. Lett..

[cit4] D'Innocenzo V., Grancini G., Alcocer M. J. P., Kandada A. R. S., Stranks S. D., Lee M. M., Lanzani G., Snaith H. J., Petrozza A. (2014). Nat. Commun..

[cit5] Miyata A., Mitioglu A., Plochocka P., Portugall O., Wang J. T. W., Stranks S. D., Snaith H. J., Nicholas R. J. (2015). Nat. Phys..

[cit6] Stranks S. D., Eperon G. E., Grancini G., Menelaou C., Alcocer M. J. P., Leijtens T., Herz L. M., Petrozza A., Snaith H. J. (2013). Science.

[cit7] Yang L. Y., Barrows A. T., Lidzey D. G., Wang T. (2016). Rep. Prog. Phys..

[cit8] Kojima A., Teshima K., Hirai Y., Miyasak T. (2009). J. Am. Ceram. Soc..

[cit9] Saliba M., Matsui T., Seo J. Y., Domanski K., Correa-Baena J. P., Nazeeruddin M. K., Zakeeruddin S. M., Tress W., Abate A., Hagfeldt A., Grätzel M. (2016). Energy Environ. Sci..

[cit10] Im J. H., Jang I. H., Pellet N., Grätzel M., Park N. G. (2014). Nat. Nanotechnol..

[cit11] Dong Q. F., Fang Y. J., Shao Y. C., Mulligan P., Qiu J., Cao L., Huang J. S. (2015). Science.

[cit12] Nie W. Y., Tsai H. H., Asadpour R., Blancon J. C., Neukirch A. J., Gupta G., Crochet J. J., Chhowalla M., Tretiak S., Alam M. A., Wang H. L., Mohite A. D. (2015). Science.

[cit13] Xiao Z. G., Dong Q. F., Bi C., Shao Y. C., Yuan Y. B., Huang J. S. (2014). Adv. Mater..

[cit14] Jeon N. J., Noh J. H., Kim Y. C., Yang W. S., Ryu S., Seok S. (2014). Nat. Mater..

[cit15] Zhang T. Y., Yang M. J., Zhao Y. X., Zhu K. (2015). Nano Lett..

[cit16] Fan P., Gu D., Liang G. X., Luo J. T., Chen J. L., Zheng Z. H., Zhang D. P. (2016). Sci. Rep..

[cit17] Ahn N., Son D. Y., Jang I. H., Kang S. M., Choi M., Park N. G. (2015). J. Am. Chem. Soc..

[cit18] Yang M. J., Zhou Y. Y., Zeng Y., Jiang C. S., Padture N. P., Zhu K. (2015). Adv. Mater..

[cit19] Ebe H., Araki H. (2016). Jpn. J. Appl. Phys..

[cit20] Zuo C. T., Ding L. M. (2014). Nanoscale.

[cit21] Zhang T. Y., Yang M. J., Zhao Y. X., Zhu K. (2015). Nano Lett..

[cit22] Bag S. T., Durstock M. F. (2016). ACS Appl. Mater. Interfaces.

[cit23] Liang Q. J., Liu J. G., Cheng Z. K., Li Y., Chen L., Zhang R., Zhang J. D., Han Y. C. (2016). J. Mater. Chem. A.

[cit24] Perumallapelli G. R., Vasa S. R., Jang J. (2016). Org. Electron..

[cit25] Zuo L. J., Dong S. Q., Marco N. D., Hsieh Y. T., Bae S. H., Sun P. Y., Yang Y. (2016). J. Am. Chem. Soc..

[cit26] Xu Q. Y., Yuan D. X., Mu H. R., Igbari F., Bao Q. L. (2016). Nanoscale Res. Lett..

[cit27] Wu W. Q., Chen D., Huang F., Cheng Y. B., Caruso R. A. (2016). J. Mater. Chem. A.

[cit28] El-Henawey M. I., Gebhardt R. S., El-Tonsya M. M., Chaudhary S. (2016). J. Mater. Chem. A.

[cit29] Wang Y. F., Li S. B., Zhang P., Liu D. T., Gu X. L., Sarvari H., Ye Z. B., Wu J., Wang Z. M., Chen Z. D. (2016). Nanoscale.

[cit30] Zhou Z. G., Huang L. M., Mei X. F., Zhao Y., Lin Z. H., Zhen H. Y., Ling Q. D. (2016). Sol. Energy.

[cit31] Huang F., Huang W. C., Dkhissi Y. M., Zhu Y., Etheridge J., Gray-Weale A., Bach U., Cheng Y. B., Spiccia L. (2014). Angew. Chem., Int. Ed. Engl..

[cit32] Liu J., Gao C., He X. L., Ye Q. Y., Ouyang L. Q., Zhuang D., Liao C., Mei J., Lau W. (2015). ACS Appl. Mater. Interfaces.

[cit33] Xiao Z. G., Dong Q. F., Bi C., Shao Y. C., Yuan Y. B., Huang J. S. (2014). Adv. Mater..

[cit34] Dinachali S. S., Bai W. B., Tu K. H., Choi H. K., Zhang J. S., Kreider M. E., Cheng L. C., Ross C. A. (2015). ACS Macro Lett..

[cit35] Li G., Shrotriya V., Huang J. S., Yao Y., Moriarty T., Emery K., Yang Y. (2005). Nat. Mater..

[cit36] Li B., Jiu T. G., Kuang C. Y., Ma S. H., Chen Q. S., Li X. D., Fang J. F. (2016). Org. Electron..

[cit37] Wang J. D., Xiang X., Yao X., Xiao W. J., Lin J., Shi W. (2016). Org. Electron..

[cit38] Wang B. B., Zhang Z. G., Ye S. Y., Rao H. X., Bian Z. Q., Huang C. H., Li Y. F. (2016). J. Mater. Chem. A.

[cit39] Liu C., Wang K., Yi C., Shi X. J., Smith A. W., Gong X., Heeger A. J. (2016). Adv. Funct. Mater..

[cit40] Zhu L. Z., Yuh B., Schoen S., Li X. P., Aldighaithir M., Richardson B. J., Alamera A., Yu Q. M. (2016). Nanoscale.

[cit41] Zhang F., Song J., Zhang L. X., Niu F. F., Hao Y. Y., Zeng P. G., Niu H. B., Huang J. S., Lian J. R. (2016). J. Mater. Chem. A.

[cit42] Sun X., Zhang C. F., Chang J. J., Yang H. F., Xi H., Lu G., Chen D. Z., Lin Z. H., Lu X. L., Zhang J. C., Hao Y. (2016). Nano Energy.

[cit43] Chen J., Shi T. F., Li X. H., Zhou B. K., Cao H. X. (2016). Appl. Phys. Lett..

[cit44] Ge Q. Q., Ding J., Liu J., Ma J. Y., Chen Y. X., Gao X. X., Wan L. J., Hu J. S. (2016). J. Mater. Chem. A.

[cit45] Yu H., Liu X. D., Xia Y. J., Dong Q. Q., Zhang K. C., Wang Z. W., Zhou Y., Song B., Li Y. F. (2016). J. Mater. Chem. A.

[cit46] Yang W. S., Noh J. H., Jeon N. J., Kim Y. C., Ryu S., Seo J., Seok S. (2015). Science.

[cit47] Saidaminov M. I., Abdelhady A. L., Murali B., Alarouse E., Burlakov V. M., Peng W., Dursun I., Wang L. F., He Y., Maculan G., Goriely A., Wu T., Mohammed O. F., Bakr O. M. (2015). Nat. Commun..

[cit48] Leijtens T., Lauber B., Eperon G. E., Stranks S. D., Snaith H. J. (2014). J. Phys. Chem. Lett..

[cit49] Jiang C. Y., Lim S. L., Goh W. P., Wei F. X., Zhang J. (2015). ACS Appl. Mater. Interfaces.

[cit50] Ma S., Shang M. W., Yu L. Y., Dong L. F. (2015). J. Mater. Chem. A.

